# Transcranial direct current stimulation over the right DLPFC selectively modulates subprocesses in working memory

**DOI:** 10.7717/peerj.4906

**Published:** 2018-05-28

**Authors:** Jiarui Wang, Jinhua Tian, Renning Hao, Lili Tian, Qiang Liu

**Affiliations:** 1 Research Center of Brain and Cognitive Neuroscience, Liaoning Normal University, Dalian, Liaoning Province, China; 2 Department of Psychology, University of Jyväskylä, Jyväskylä, Finland

**Keywords:** Right DLPFC, tDCS, Working memory, *n*-back, Maintenance, Updating

## Abstract

**Background:**

Working memory, as a complex system, consists of two independent components: manipulation and maintenance process, which are defined as executive control and storage process. Previous studies mainly focused on the overall effect of transcranial direct current stimulation (tDCS) on working memory. However, little has been known about the segregative effects of tDCS on the sub-processes within working memory.

**Method:**

Transcranial direct current stimulation, as one of the non-invasive brain stimulation techniques, is being widely used to modulate the cortical activation of local brain areas. This study modified a spatial *n*-back experiment with anodal and cathodal tDCS exertion on the right dorsolateral prefrontal cortex (DLPFC), aiming to investigate the effects of tDCS on the two sub-processes of working memory: manipulation (updating) and maintenance. Meanwhile, considering the separability of tDCS effects, we further reconfirmed the causal relationship between the right DLPFC and the sub-processes of working memory with different tDCS conditions.

**Results:**

The present study showed that cathodal tDCS on the right DLPFC selectively improved the performance of the modified 2-back task in the difficult condition, whereas anodal tDCS significantly reduced the performance of subjects and showed an speeding-up tendency of response time. More precisely, the results of discriminability index and criterion showed that only cathodal tDCS enhanced the performance of maintenance in the difficult condition. Neither of the two tDCS conditions affected the performance of manipulation (updating).

**Conclusion:**

These findings provide evidence that cathodal tDCS of the right DLPFC selectively affects maintenance capacity. Besides, cathodal tDCS also serves as an interference suppressor to reduce the irrelevant interference, thereby indirectly improving the working memory capacity. Moreover, the right DLPFC is not the unique brain regions for working memory manipulation (updating).

## Introduction

Transcranial direct current stimulation (tDCS), as one of the non-invasive brain stimulation techniques, have been widely used to modulate the cortical excitability of local brain areas by applying safe and weak direct current on the scalp, with its impact persisting for a varied period of time depending on the current intensity and duration. It has been demonstrated that anodal stimulation of tDCS in motor cortex would depolarize the resting-membrane potential of cortex area under the stimulating electrode, leading to the facilitation of related functions. On the other hand, cathodal stimulation would hyperpolarize the resting-membrane potential to suppress excitability, which is known as a suppressive effect (Nitsche et al., 2003, 2004, 2007, 2009; Nitsche & Paulus, 2000). With the above characteristics, tDCS allows us to explore the underlying mechanism within sophisticated cognitive process and the causal relationship between functions and relevant cortex (de Graaf & Sack, 2014). In addition, tDCS has also been used as a therapeutic method to relieve symptoms in patients with cognitive deficits. Moreover, tDCS has also been used among healthy populations to enhance cognitive performance (Kuo & Nitsche, 2015; Sandrini & Cohen, 2013; Wang et al., 2016).

Working memory, as the most crucial foundation of cognitive processes, is defined as a dynamical memory system that can temporarily manipulate and store the information online (Baddeley, 2007). A great number of experiments have investigated how tDCS modulates working memory capacity and they reported the overall effects of tDCS stimulation on working memory performance (Hill, Fitzgerald & Hoy, 2016). However, working memory is a rather complex system. As most definitions describes, working memory can be divided into two relatively independent functional components: executive control and storage process (Shah & Miyake, 1999). Executive control refers to the manipulation and regulation processes of working memory contents. Afterwards, the storage process gets involved in the active maintenance of a limited capacity of information for a few seconds, which is a necessary component to serve the executive control. Although executive control operates on the contents of the storage process, these two components of working memory are relatively independent. This claim has been proved by the emerging evidence that neurological patients who had intact short-term storage but defective executive processes and vice versa (D’Esposito & Postle, 2000). However, due to the lack of experimentation, little has been revealed about the segregative effects of tDCS stimulation on the manipulation and maintenance within working memory. The working memory system resembles a computer. Either the improvement of processing power or the increase of processing capacity could enhance its overall performance. Thus, it is necessary to investigate how the sub-processes modulate the overall performance of working memory with tDCS stimulation and to explore the specific effect of tDCS stimulations on the sub-processes.

According to previous definitions, executive control is an overarching term containing diverse cognitive sub-functions, which can be decomposed into several individual functions, such as “updating,” “inhibition of prepotent response” and “attention shifting” (Hofmann, Schmeichel & Baddeley, 2012; Miyake et al., 2000). Of the above functions, “updating” is a crucial executive function which is responsible for the continuously replacing outdated representation with new information, and meanwhile abandoning the unwanted messages (Baddeley, 2007; Kane et al., 2001). According to previous experiments, the *n*-back task is a flexible and widely used experimental paradigm for measuring memory updating function and working memory capacity. In the task, participants were required to monitor a continuous sequence of stimulus and recall whenever the presented stimulus was the same as the “*n*” trial presented before, with “*n*” usually set as 0, 1, 2 (Owen et al., 2005). With the increase of cognitive demands in different “*n*” conditions, the performance of sub-processes within working memory can be detected. In the experiment, 0-back task served as a control condition, which required participants to respond whether the present stimulus was the same with the first pre-specified stimulus. The 0-back condition does not require any representation manipulation of working memory. Compared with the 0-back task, 1-back task involves the manipulation process which is defined as updating. The 1-back task is viewed as pure measurement of updating function (Szmalec et al., 2009). Compared with the 1-back task, 2-back task involves both manipulation (updating) and maintenance process, in which participants were required to maintain the present stimulus for a moment. In summary, *n*-back task involves updating function and maintenance for storing different materials, which can be represented as two relatively independent systems, namely executive control and storage process. Therefore, *n*-back paradigm can be employed to testify our hypothesis that tDCS effects can induce different effects on the sub-processes of working memory.

Functional neuroimaging studies have demonstrated that working memory is highly correlated with the activity in dorsolateral prefrontal cortex (DLPFC) (Curtis, 2006; Curtis & D’Esposito, 2003; Ku et al., 2007; Postle, 2006) and posterior parietal cortex (PPC) (Ku et al., 2015b; Todd & Marois, 2005; Vogel, McCollough & Machizawa, 2005; Xu & Chun, 2005). Furthermore, executive control and storage process are mediated partially by the prefrontal cortex (D’Esposito et al., 1999; Curtis, 2006; Ku, Bodner & Zhou, 2015; Owen, 1997). In particular, the dorsolateral frontal cortex is crucially involved in the sub-processes of working memory, including the suppression of distraction (Anderson et al., 2004), updating function (Toepper et al., 2010), and on-line maintenance of representation (Courtney et al., 1998; Zhao et al., 2017). A meta-analysis of functional magnetic resonance imaging in *n*-back tasks demonstrated that specific activation related to updating was specifically at right-lateral frontal area (McKenna, Rushe & Woodcock, 2017). Therefore, the right DLPFC tends to be the region of interest, which involves both manipulation (updating) and maintenance.

However, very few studies have investigated tDCS effects over the right DLPFC (Giglia et al., 2014; Salehinejad, Rostami & Ghanavati, 2015; Wu et al., 2016). A controversial phenomenon has been observed that tDCS effects on changing working memory ability were not always clear and polarity-specific on the right DLPFC. It might be the results of multiple underlying mechanisms involved in the right DPFLC that served for working memory performance. The right DLPFC has been regarded to play a specific role in attention control, which was closely related to selective attention and maintenance of task-relevant information (Li et al., 2017). Meanwhile, it was also important for inhibition control, such as suppression of inappropriate responses (Aron, Robbins & Poldrack, 2014; Li et al., 2017). It has been shown that cathodal tDCS over the right DLPFC could enhance recognition memory performance by suppressing the interference in the task (Smirni et al., 2015). Therefore, both anodal and cathodal tDCS of the right DLPFC might have the potential to improve working memory: the anodal tDCS could enhance the updating and maintaining ability of task-relevant information based on the tDCS effects of enhancing anodal and inhibiting cathodal patterns, while the cathodal tDCS might have promotive effects on the sub-processes of working memory as an interference suppressor. The above results converged to show that there might be a major path for tDCS to regulate the performance of working memory by employing different tDCS conditions.

To sum up, although extensive tDCS experiments have discussed the overall effects of tDCS on working memory performance, they failed to provide sufficient evidence to illustrate which sub-processes of working memory were affected by anodal/cathodal tDCS stimulation. These studies (Li et al., 2017; Oliveri et al., 2001) also pointed out that it was necessary to examine the underlying mechanism of tDCS effects, and investigate cognitive sub-processes and neural correlates associated with different phases of working memory. Therefore, the current experiment aimed to investigate segregative effects of tDCS on the sub-processes of working memory. We hypothesized that the performance of each sub-process of working memory, namely manipulation (updating) and maintenance, might be affected independently by tDCS stimulation, and we could observe a more elaborate causal relationship between the right DLPFC and the sub-processes with different tDCS conditions. Furthermore, the current study aims to reconfirm the main underlying mechanism of the right DLPFC that tDCS could directly modulate the performance of working memory with different tDCS conditions.

## Methods

### Participants

Thirty right-handed healthy subjects (15 males, 15 females) participated in this study. The age range was 23.2 ± 1.9 years (a predominantly student-based sample). All participants reported a normal or corrected-to-normal vision. One subject was excluded from data analysis due to low accuracy of behavioral response in the experiment. All participants had no metallic implant and history of neurological impairment or psychiatric diagnoses. Participants were not aware of the tDCS purpose and procedure applied throughout the testing phase.

All procedures performed in studies involving human participants were in accordance with the ethical standards of the institutional and/or national research committee and with the 1964 Helsinki Declaration and its later amendments or comparable ethical standards. The study was approved by Human Research Institutional Review Board at Liaoning Normal University (the approval number: lNNUIRB1710). Methods were carried out in accordance with relevant guidelines. Written informed consents were collected from participants.

### Materials and design

A series of stimuli were presented to the participants continuously. Each trial was presented for 500 ms and followed by a 3,000 ms blank for the delay interval, then followed by the next trial. In each trial, a blue square with a subtended 2.6° × 2.6° visual angle was presented on the gray screen, which randomly appeared in 24 positions around the central gaze point in a square range with a 14° × 14° visual angle. Stimuli were presented by E-Prime 2.0 (Psychology Software Tools, Pittsburgh, PA, USA). All subjects were required to sit in front of a 40 cm × 25 cm monitor with a viewing distance of approximate 60 cm. Participants were required to remember the position of the blue square and determine whether the position was the same according to different “*n*” back tasks. Participants were required to respond as quickly and accurately as possible and simultaneously avoid verbal memory of position.

A modified *n*-back task was adopted with the position as variable. *N* was manipulated in three conditions: 0, 1 and 2. The 0-back experiment required participants to judge whether the position of current stimulus was the same as the first trial of the series of stimuli. The 1-back experiment required participants to respond whether the position of the current stimuli was the same with that of the previous trial. Similarly, in the 2-back experiment, participants needed to compare the position of the current stimuli with that presented two trials before.

**Figure 1:**
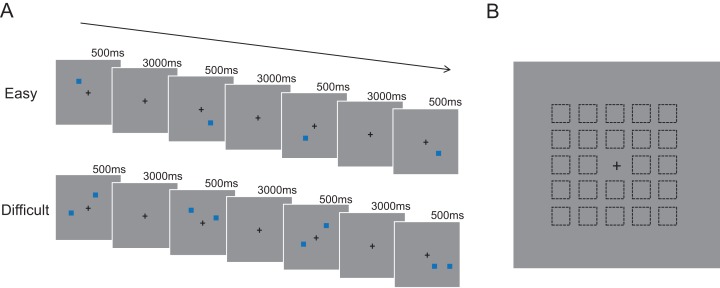
Experimental materials and procedures. (A) Experimental procedure used in our modified *n*-back task. (B) All positions that blue squares appear. (Dotted box didn’t exist during the experimentation).

The *n*-back paradigm was also modified by manipulating perceptual loads with two difficulty conditions, namely, easy condition and difficult condition. In the easy condition, participants were required to compare the position of one square. In the difficult condition, participants needed to remember and compare the position of two squares. Participants were supposed to press “yes” when either of the presented positions was identical to the previous stimulus. For both difficulty conditions, there were in total 12 blocks randomly presented in the sequential experimental stages with each four blocks of “*n* = 0,” “*n* = 1,” “*n* = 2” condition (22 trials for each block). Participants were given enough time between each blocks to relax and get ready to start. Before the formal experiment, all participants were required to get familiar with the requirements in the practice phase. The practice phase consisted of two blocks for each condition and 15 trials within each block (Fig. 1).

### tDCS

The instrument employed in the current research is ActivaDose®II (ActivaTek Inc., Gilroy, CA, USA). The electrodes consisted of two saline-soaked surface sponge electrodes (5 × 5 cm^2^) plus conductive iron tablets. There were three stimulation conditions: anodal, cathodal and sham. During the period of applying tDCS, the size of anodal (cathodal) stimulus was the constant current of 1.5 mA, lasting for 20 min before the task. Based on previous methods, one electrode was placed on the right DLPFC (F4) in all conditions according to the international 10–20 EEG system. The contralateral supraorbital area was chosen as the reference electrode position (Fregni et al., 2005) for the reason that this area was functionally irrelevant to working memory and less likely to affect memory performance. Since the stimulation had a 30 s linear fade time with the current slowly varying from 0 to 1.5 mA, participants would feel an itching sensation at the beginning of anodal (cathodal) condition. The sham stimulation was set as 0.5 mA with duration of 2 min, and the duration of fade time was same with anodal (cathodal) stimulus to keep the same initial feelings between different tDCS conditions.

### Procedure

The experiment consisted of two sessions. In the first session, all participants were required to complete the modified *n*-back task without tDCS stimulation. In the second session, participants were randomly divided into three groups to receive different tDCS stimulation (anodal, cathodal and sham), respectively. Afterward, they were asked to perform the same *n*-back tasks. The order of the two difficulty conditions was counterbalanced in the second session. Sufficient interval time (about one week) was set between the two sessions to eliminate practice effect from the first session. Participants were comfortably seated in a moderately lit, sound attenuated and electrically shielded experimental room. The two sessions were conducted simultaneously at the same time and place to make ensure that participants had the same mental state.

### Data analysis

#### Baseline

A 2 × 3 repeated measures ANOVA of hit rate and response time with tDCS difficulty (easy vs. difficult) and “*n*” condition (0 vs. 1 vs. 2) for baseline was conducted to verify the validity of the modified *n*-back experiments and summarize behavior characteristic of participants. The *p* values of ANOVA were corrected with Greenhouse–Geisser adjustment when the sphericity assumption was violated and post-hoc pairwise comparisons were corrected with Sidak adjustment.

#### Hit rate and response time of each tDCS condition

We analyzed the hit rate and response time of each tDCS conditions (anodal, cathodal, sham), in order to observe the trend of initial changes in different “*n*” conditions. We compared each tDCS condition with its baseline in all factors. Totally 3 three-way repeated measures ANOVA of hit rate and response time were conducted with tDCS stimulation (baseline vs. stimulation), difficulty (easy vs. difficult) and “*n*” condition (0 vs. 1 vs. 2) as within-subject measures. Post-hoc analysis and pairwise comparisons were also performed to explore statistically significant interactions. The *p* values of ANOVA were corrected with Greenhouse–Geisser adjustment when the sphericity assumption was violated and post-hoc pairwise comparisons were corrected with Sidak adjustment.

#### Difference of each stimulation conditions minus baseline

To confirm the tDCS effects, we mainly analyzed the difference by subtracting baseline value of hit rate and response time from that of each tDCS stimulation conditions. Then, a mixed 2 × 3 × 3 ANOVA was conducted, with tDCS condition (anodal vs. sham and cathodal vs. sham) as a between-subject factor, the difficulty (easy vs. difficult), and “*n*” condition (0 vs. 1 vs. 2) as within-subject factors. Post-hoc analysis was performed to explore statistically significant interactions. The *p* values of ANOVA were corrected with Greenhouse–Geisser adjustment when the sphericity assumption was violated. Furthermore, independent sample *t* test was conducted to explore the difference between anodal and sham stimulation, cathodal and sham stimulation in all within-subject factors’ conditions. Statistical power was executed by G-Power 3.1.

#### Discriminability index (d′) and criterion (β) of each tDCS condition

The discriminability index (*d*′) and criterion (β) were two independent indicators of signal detection theory, which were used to separate sensory and non-sensory factors (MacMillan, 2002). The discriminability index (*d*′) was a parameter to measure the sensitivity of decision-making process. The discriminability index (*d*′) was calculated from the *z* scores of the hit rate and the false alarm rate and the formula is *d*′ = *z*(hit rate) − *z*(false alarm rate). The criterion (β) was an indicator used to indicate response bias related to subjective factors of participants, such as motivation and attitude. β was the ratio of the ordinate of the hit rate distribution at the criterion to the ordinate of the false alarm rate distribution at the criterion. Therefore, the formula can be expressed as β = *o*(hit rate)/*o*(false alarm rate). The value of β can be found in the table of criterion values (β) (Gardner et al., 1984).

A three-way repeated measures ANOVA of discriminability index (*d*′) and criterion (β) was conducted, respectively in three tDCS condition (anodal, cathodal, sham) with tDCS stimulation (baseline vs. stimulation), difficulty (easy vs. difficult) and “*n*” condition (0 vs. 1 vs. 2) as within-subject factor. Post-hoc analysis and pairwise comparison were performed to explore statistically significant effects in all conditions. The *p* values of ANOVA were corrected with Greenhouse–Geisser adjustment when the sphericity assumption was violated and post-hoc pairwise comparisons were corrected with Sidak adjustment.

## Results

### Pre-stimulation effects

**Figure 2:**
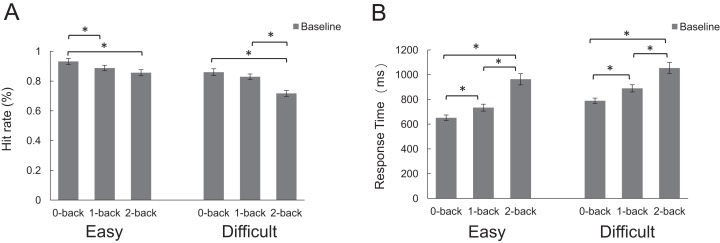
The hit rate and response time of pre-stimulation effects in baseline. (A) The hit rate. (B) The response time. (*p < 0.05).

Baseline. A 2 × 3 repeated measures ANOVA of hit rate in baseline revealed the main effect of difficulty (*F*_1,28_ = 11.36, *p* = 0.002, *η_p_*^2^ = 0.28) and “*n*” condition (*F*_2,56_ = 41.54, *p* < 0.001, *η_p_*^2^ = 0.59). The interaction between difficulty and “*n*” condition (*F*_2,56_ = 5.18, *p* = 0.009, *η_p_*^2^ = 0.15) was also significant, which indicated that the hit rate was significantly decreased upon the increase in difficulty and “*n.*” Post-hoc analysis revealed that in the easy condition (0-back: *M* = 0.923, *SD* = 0.116; 1-back: *M* = 0.878, *SD* = 0.112; 2-back: *M* = 0.842, *SD* = 0.119), the performance of 0-back task was significantly better than that of 1-back (*p* = 0.024) and 2-back task (*p* = 0.001). No significant difference was found between 1-back and 2-back tasks (*p* = 0.130). In the difficult condition (0-back: *M* = 0.859, *SD* = 0.123; 1-back: *M* = 0.828, *SD* = 0.101; 2-back: *M* = 0.716, *SD* = 0.108), there was no significant difference between 0-back and 1-back (*p* = 0.313), but a significant difference between 1-back and 2-back, and 0-back was significantly different with 2-back (both *p* < 0.001). Another 2 × 3 repeated measures ANOVA revealed statistically significant effect of difficulty (*F*_1,28_ = 42.87, *p* = 0.000, *η_p_*^2^ = 0.60) and “*n*” condition (*F*_2,56_ = 41.25, *p* < 0.001, *η_p_*^2^ = 0.59) (In the easy condition: 0-back: *M* = 651.68, *SD* = 122.14; 1-back: *M* = 733.73, *SD* = 149.97; 2-back: *M* = 963.35, *SD* = 245.33; In the difficult condition: 0-back: *M* = 789.70, *SD* = 114.71; 1-back: *M* = 889.84, *SD* = 156.67; 2-back: *M* = 1054.59, *SD* = 242.84), and no interaction (*F*_2,56_ = 1.80, *p* = 0.174) between difficulty and “*n*” condition on response time (Fig. 2).

### tDCS effects

#### Hit rate

##### Anodal stimulation

The main effects of tDCS stimulation (*F*_1,9_ = 5.19, *p* = 0.049, *η_p_*^2^ = 0.36), “*n*” condition (*F*_2,18_ = 13.75, *p* = 0.000, *η_p_*^2^ = 0.60), and difficulty (*F*_1,9_ = 11.39, *p* = 0.008, *η_p_*^2^ = 0.55) were significant. No interaction effect was observed among the three factors (*F*_2,18_ = 0.92, *p* = 0.41). A marginally significant interaction of difficulty and tDCS stimulation was observed (*F*_1,9_ = 2.26, *p* = 0.16). Pairwise comparison revealed that only in the difficult condition (stimulation: *M* = 0.760, *SD* = 0.044; baseline: *M* = 0.804, *SD* = 0.033), anodal stimulation would decrease the hit rate significantly (*p* = 0.042), which was only shown in the 2-back experiment (stimulation: *M* = 0.660, *SD* = 0.052; baseline: *M* = 0.735, *SD* = 0.041; *p* = 0.039). (*n* = 0: *p* = 0.528; *n* = 1: *p* = 0.154).

##### Cathodal stimulation

The main effects of “*n*” condition (*F*_2,16_ = 32.02, *p* = 0.000, *η_p_*^2^ = 0.80), difficulty (*F*_1,8_ = 15.82, *p* = 0.004, *η_p_*^2^ = 0.66) and a marginally significant tDCS stimulation effect (*F*_1,8_ = 4.70, *p* = 0.062) were observed. In addition, interactions of “*n*” condition × tDCS stimulation (*F*_2,16_ = 4.22, *p* = 0.034, *η_p_*^2^ = 0.346), difficulty × tDCS stimulation (*F*_1,8_ = 15.34, *p* = 0.004, *η_p_*^2^ = 0.657) and among three factors (*F*_2,16_ = 4.13, *p* = 0.036, *η_p_*^2^ = 0.34) were significant. Post-hoc analysis indicated that in the 2-back condition (stimulation: *M* = 0.863, *SD* = 0.026; baseline: *M* = 0.803, *SD* = 0.021; *F*_1,8_ = 11.73, *p* = 0.009), the hit rate was significantly increased in the cathodal stimulation. And the hit rate was also significantly increased in the difficult condition (stimulation: *M* = 0.848, *SD* = 0.032; baseline: *M* = 0.797, *SD* = 0.037; *F*_1,8_ = 10.64, *p* = 0.011). Furthermore, pairwise comparison revealed that only in the difficult condition of 2-back experiment (stimulation: *M* = 0.820, *SD* = 0.035; baseline: *M* = 0.702, *SD* = 0.034), cathodal stimulation would induce a significant increase of hit rate (*p* = 0.000).

##### Sham stimulation

The main effects of “*n*” condition (*F*_2,18_ = 9.27, *p* = 0.002, *η_p_*^2^ = 0.51) and difficulty (*F*_1,9_ = 7.89, *p* = 0.02, *η_p_*^2^ = 0.46) were observed. Neither the main effect of tDCS stimulation (*F*_1,9_ = 0.06, *p* = 0.799) nor the interactions among three factors were significant (*F*_1,9_ = 1.28, *p* < 0.30).

##### Difference of hit rate between tDCS conditions

**Figure 3:**
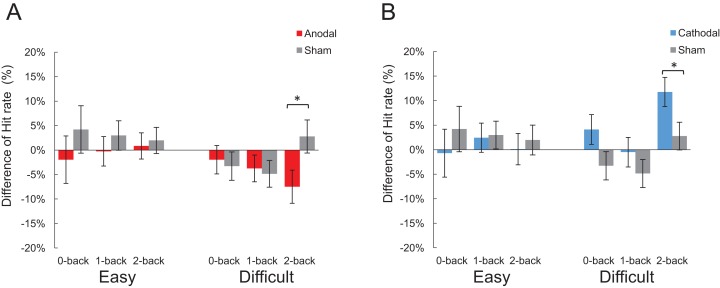
Comparison of each stimulation conditions minus their baseline in hit rate. (A) Anodal tDCS compared with sham tDCS. (B) Cathodal tDCS compared with sham tDCS. (*p < 0.05).

A mixed 2 × 3 × 3 repeated measures ANOVA of the hit rate difference among anodal, cathodal and sham conditions minus their baseline revealed no significant main effect for “*n*” condition (*F*_2,52_ = 2.51, *p* = 0.09), difficulty (*F*_1,26_ = 1.52, *p* = 0.229) and a marginally significant tDCS condition effect (*F*_2,26_ = 2.721, *p* = 0.085). The interaction effect of difficulty × tDCS condition was significant (*F*_2,26_ = 3.23, *p* = 0.046, *η_p_*^2^ = 0.15), and post-hoc analysis revealed that the tDCS effects significantly affected the hit rate in the difficult condition (anodal: *M* = −0.043, *SD* = 0.021; cathodal: *M* = 0.051, *SD* = 0.022; sham: *M* = −0.018, *SD* = 0.021; *F*_2,26_ = 5.10, *p* = 0.013), not the easy condition (*F*_2,26_ = 1.69, *p* = 0.203). Furthermore, independent sample *t* test revealed that in the difficult condition, anodal stimulation significantly decreased the hit rate (anodal: *M* = −0.074, *SD* = 0.097; sham: *M* = −0.027, *SD* = 0.115; *t*_18_ = 2.141, *p* < 0.05, Cohen’s *d* = 0.44) in the 2-back rather than 0-back (anodal: *M* = −0.01, *SD* = 0.094; sham: *M* = −0.032, *SD* = 0.088; *t*_18_ = 0.325, *p* > 0.05) or 1-back (anodal: *M* = −0.037, *SD* = 0.076; sham: *M* = −0.048, *SD* = 0.095; *t*_18_ = 0.285, *p* > 0.05), compared to sham stimulation. In addition, a significant increase was observed in cathodal stimulation in the 2-back (cathodal: *M* = 0.117, *SD* = 0.044; sham: *M* = −0.027, *SD* = 0.115; *t*_17_ = 2.186, *p* < 0.05, Cohen’s *d* = 1.65), but not the 0-back (cathodal: *M* = 0.04, *SD* = 0.093; sham: *M* = −0.032, *SD* = 0.088; *t*_17_ = 1.765, *p* > 0.05) or 1-back task (cathodal: *M* = −0.005, *SD* = 0.084; sham: *M* = −0.048, *SD* = 0.095; *t*_17_ = 1.045, *p* > 0.05), compared to the sham stimulation in the difficult condition (Fig. 3).

#### Response time

##### Anodal stimulation

The main effects of tDCS stimulation (*F*_1,9_ = 24.78, *p* = 0.001, *η_p_*^2^ = 0.73), “*n*” condition (*F*_2,18_ = 13.41, *p* = 0.000, *η_p_*^2^ = 0.59), and difficulty (*F*_1,9_ = 21.00, *p* = 0.001, *η_p_*^2^ = 0.70) were observed. The interaction between “*n*” condition and tDCS stimulation was significant (*F*_2,18_ = 4.48, *p* = 0.026, *η_p_*^2^ = 0.33). Post-hoc analysis revealed that participants trended to respond faster after anodal stimulation in all “*n*” condition (0-back: stimulation: *M* = 624.01, *SD* = 43.54; baseline: *M* = 692.17, *SD* = 39.04; *p* = 0.019; 1-back: stimulation: *M* = 699.03, *SD* = 42.16; baseline: *M* = 808.34, *SD* = 60.90; *p* = 0.004; 2-back: stimulation: *M* = 808.14, *SD* = 77.38; baseline: *M* = 1000.32, *SD* = 86.52; *p* = 0.003).

##### Cathodal stimulation

Significant main effects of “*n*” condition (*F*_2,16_ = 11.38, *p* = 0.001, *η_p_*^2^ = 0.58) and difficulty conditions (*F*_1,8_ = 58.37, *p* = 0.000, *η_p_*^2^ = 0.87) were observed. Neither the main effect of tDCS stimulation (*F*_1,8_ = 2.96, *p* = 0.123, *η_p_*^2^ = 0.27) nor the interaction between all factors were significant.

##### Sham stimulation

The main effects of tDCS stimulation (*F*_1,9_ = 5.49, *p* = 0.044, *η_p_*^2^ = 0.379), “*n*” condition (*F*_2,18_ = 28.29, *p* = 0.000, *η_p_*^2^ = 0.75), and difficulty (*F*_1,9_ = 28.24, *p* = 0.000, *η_p_*^2^ = 0.75) were observed. No interaction effect was observed among the three factors (*F*_2,18_ = 0.26, *p* = 0.77). Pairwise comparison revealed that participants’ response became faster in the easy condition (0-back: stimulation: *M* = 603.80, *SD* = 31.23; baseline: *M* = 707.04, *SD* = 27.82; 1-back: stimulation: *M* = 644.19, *SD* = 41.46; baseline: *M* = 776.42, *SD* = 36.78) in the 0-back (*p* = 0.005) and 1-back (*p* = 0.005) task due to practice effects.

##### Difference of response time between tDCS conditions

**Figure 4:**
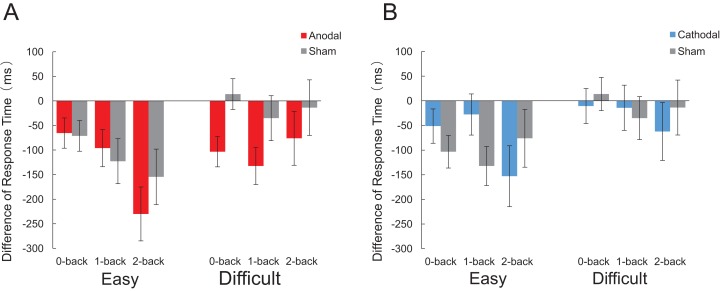
Comparison of each stimulation conditions minus their baseline in response time. (A) Anodal tDCS compared with sham tDCS. (B) Cathodal tDCS compared with sham tDCS. (*p < 0.05).

A mixed 2 × 3 × 3 repeated measures ANOVA of response time difference revealed main the effect of “*n*” condition (*F*_2,52_ = 4.01, *p* = 0.024, *η_p_*^2^ = 0.134), and difficulty (*F*_1,26_ = 5.71, *p* = 0.024, *η_p_*^2^ = 0.180), but not tDCS condition (*F*_2,26_ = 2.198, *p* = 0.131). A marginal interaction effect of “*n*” condition and tDCS condition was observed (*F*_2,26_ = 1.938, *p* = 0.11) and the response time tend to decreased in the 2-back task (*p* = 0.088) with anodal tDCS (Fig. 4).

##### Discriminability index (d′) and criterion (β) of each tDCS condition

The discrimination (*d*′) and criterion (β) in each tDCS conditions were analyzed, respectively. The main effect of tDCS stimulation was not significant in anodal condition (*F*_1,9_ = 0.02, *p* = 0.88) and sham condition (*F*_1,9_ = 0.08, *p* = 0.78). Only in the cathodal condition, the main effect of tDCS stimulation was significant (*F*_1,9_ = 11.19, *p* = 0.009, *η_p_*^2^ = 0.55). Pairwise comparison revealed that only in the difficult condition in the 2-back task (stimulation: *M* = 2.025, *SD* = 0.156; baseline: *M* = 1.539, *SD* = 0.201), the discrimination (*d*′) increased across two sessions (*p* = 0.006). The analysis of criterion (β) showed that only in the anodal condition, the mainly effect of tDCS stimulation reached a marginal significance (*F*_1,9_ = 3.89, *p* = 0.08). No significant main effect of tDCS stimulation was observed in cathodal condition (*F*_1,9_ = 1.29, *p* = 0.28) and sham condition (*F*_1,9_ = 2.39, *p* = 0.15). Further analysis revealed that only in the difficult condition in the 2-back task (stimulation: *M* = 2.542, *SD* = 0.636; baseline: *M* = 1.933, *SD* = 0.700), the criterion (β) was decreased across two sessions (*p* = 0.020) in the anodal condition (Table 1).

**Table 1:** The discriminability index (*d*′) and Response bias (β) in anodal and cathodal conditions.

	0-back	1-back (easy)	2-back	0-back	1-back (difficult)	2-back
**Discriminability index (*d*′)**						
Baseline	3.58 (±0.176)	3.36 (±0.162)	2.62 (±0.240)	2.12 (±0.199)	2.33 (±0.204)	1.68 (±0.165)
Anodal	3.23 (±0.200)	3.34 (±0.193)	2.91 (±0.236)	2.13 (±0.211)	2.36 (±0.183)	1.80 (±0.164)
Baseline	3.47 (±0.145)	3.44 (±0.170)	2.97 (±0.192)	2.26 (±0.275)	2.31 (±0.238)	1.54 (±0.201)
Cathodal	3.56 (±0.214)	3.74 (±0.184)	3.31 (±0.203)	2.46 (±0.200)	2.35 (±0.152)	2.03 (±0.156)
**Response bias (β)**						
Baseline	0.97 (±0.214)	2.40 (±0.472)	1.70 (±0.435)	0.81 (±0.177)	1.71 (±0.287)	1.93 (±0.700)
Anodal	0.86 (±0.194)	2.81 (±0.631)	2.25 (±0.390)	0.91 (±0.131)	2.77 (±0.864)	2.54 (±0.637)
Baseline	0.68 (±0.159)	2.61 (±0.713)	2.08 (±0.472)	0.62 (±0.145)	1.68 (±0.331)	1.66 (±0.327)
Cathodal	1.09 (±0.284)	2.78 (±0.586)	3.00 (±0.727)	0.64 (±0.180)	1.86 (±0.398)	1.20 (±0.313)

## Discussion

The current study mainly examined the segregative effect of tDCS stimulation on the sub-processes, manipulation (updating) and maintenance of working memory by employing a spatial *n*-back task. The *n*-back paradigm was widely used to modulate working memory capacity with tDCS technique. However, it is still unclear whether these effects reflect an enhancement in the executive control (updating) or maintenance, since both of them contribute to working memory performance. The current study further discussed the mechanism of tDCS effects by clearly dissociating the executive control (updating) from maintenance. With the manipulation of three “*n*” conditions and two types of perceptual loads, the modified *n*-back paradigm effectively separated the updating and maintaining process. Meanwhile, the 1-back task served as another measuring index of updating to reconfirm the tDCS effects on executive control. With the tDCS stimulation of the right DLPFC, it was observed that tDCS resulted in different effects on the two sub-processes: manipulation (updating) and maintenance, respectively. Cathodal tDCS facilitated the maintenance of working memory, while neither anodal nor cathodal tDCS exerted influence on the performance of updating. The detailed results are discussed below.

The present study found that tDCS stimulation only induced significant changes of working memory performance in the difficult condition. Several tDCS studies have demonstrated that the tDCS effects was closely related to task difficulty and only in the high difficulty condition could tDCS induce a significant behavioral change (Pope, Brenton & Miall, 2015; Roe et al., 2016; Wu et al., 2014). Based on the conventional *n*-back paradigm, the current study introduced perceptual loads to provide hierarchical difficulty for tDCS to modulate performance effectively. Pre-stimulation results has proved the validity of the modified paradigm and meanwhile provided direct evidence that updating and maintenance processes can be dissociated in the modified *n*-back task. In the easy condition, a significant difference of hit rate was observed between 1-back and 0-back task, which might be the result of manipulation (updating). No significant difference between 1-back and 2-back task was observed concerning maintaining process. It was therefore inferred that the hit rate difference was mainly caused by updating rather than maintenance, since it was easy for participants to keep one square in mind. Therefore, it can be inferred that the difference of hit rate in behavior was mainly caused by updating in the easy condition. In the difficult condition, a significant hit rate difference was caused by maintenance process between 1-back and 2-back, but no difference was revealed between 0-back and 1-back. Due to the difficulty of two squares, participants tended to allocate more cognitive resource on maintaining information. Consequently, the updating process would be relatively less effort-consuming and easy to induce significant difference of hit rate, compared with maintenance process. Thus, in the difficult condition, the difference of hit rate can be mainly accounted by maintenance. In addition, response time significantly increased both in the easy and difficult condition. According to previous studies, the right DLPFC was closely related to manipulation and maintenance of working memory, such as sub-functions of executive control (updating) and on-line maintenance of information (D’Esposito et al., 1999; Courtney et al., 1998; Curtis, 2006; Owen, 1997; Toepper et al., 2010; Zhao et al., 2017). Results of the current study showed that tDCS significantly induced behavioral changes in the maintenance rather than the manipulation process in the difficult condition. More specifically, cathodal tDCS improved the performance of maintenance of working memory. In contrast, anodal tDCS of the right DLPFC significantly decreased the hit rate and shortened the response time in the difficult condition.

In the current study, both anodal and cathodal tDCS failed to induce any effect on the executive control process (updating). In Toepper et al. (2010) research, it was claimed that the DLPFC was crucial for higher-level executive processing, such as updating information and suppressing distractions. Besides, McKenna, Rushe & Woodcock (2017) demonstrated that the specific activation area associated with updating was right-lateralized frontal region. Therefore, it can be speculated that the fluctuation of activation over the right DLPFC would induce differences in terms of updating performance. However, our results suggested that there was no direct causal relationship between updating and the right DLPFC. This claim seemed to be consistent with previous studies which attempted to isolate the updating-specific process from executive control with functional and morphometric index of brain networks (Smolker et al., 2015). It was proposed that the updating process may rely more on a specific area involved in working memory and less on the connectivity between areas. Thus, the tDCS of the right DLPFC failed modulate the updating performance directly.

An interesting result in our study was the reversal polarity effects observed with tDCS exertion on the right DLPFC: Cathodal tDCS facilitated the maintenance of working memory and anodal tDCS showed a suppressive effect. The typical pattern of anodal enhancement and cathodal inhibition seems not applicable to the relationship between the right DLPFC and working memory. Due to the direct relationship between working memory ability and its related functional cortex, previous studies observed the enhancing anodal and inhibiting cathodal patterns of tDCS effects on working memory ability with the left DLPFC as the stimulation electrode position (Hill, Fitzgerald & Hoy, 2016; Liu et al., 2017; Pope, Brenton & Miall, 2015; Shen et al., 2016). However, in our results, it was suggested that the right DLPFC may play another role in working memory. The right DLPFC was regarded to play a critical role in attention control, which was tightly related to the maintenance of task-relevant information and inhibition of task-irrelevant information (Li et al., 2017). In Li et al.’s study, it was also observed that tDCS of the right DLPFC significantly modulated the performance of visual working memory in the distractor condition. A few recent studies exploring tDCS-related modulation of the right DLPFC also showed similar reversal results as the present study. For instance, cathodal tDCS over the right DLPFC was reported to improve the performance of non-verbal recognition memory significantly (Smirni et al., 2015). It was interpreted that cathodal tDCS provided a more appropriate activation for the inhibition control of the right DLPFC, instead of the anodal tDCS. At the neural level, it was suggested that cathodal tDCS might cause a depression of cortical inhibitory interneurons and this disinhibition would indirectly enhance the function of the stimulated cortex (Monti et al., 2008). Through a combinatory employment of tDCS and EEG recordings, Zaehle et al. (2011) found a decreased alpha oscillation with an enhanced WM performance in *n*-back task after cathodal tDCS stimulation over the DLPFC. Alpha oscillations have been reported to reflect general inhibition of non-task relevant areas (Klimesch et al., 2000) and closely relate to the suppression of activation representing task-irrelevant information. Moreover, alpha oscillations serve as an index of inhibition degree of internal attention (Cooper et al., 2003). Heimrath et al. (2012) have demonstrated that during the retention of visuo-spatial information, cathodal tDCS of the right parietal cortex would induce a facilitative effect on the working memory manifested in a decreased alpha oscillation. The above results provided supportive evidences for the assumption that cathodal tDCS modulates inhibition control in terms of task-irrelevant information, which would indirectly enhance the maintenance performance of working memory.

In the majority of tDCS studies, it can be seen that tDCS stimulation often influenced a wider range of area than the exact regions of interest. Therefore, the imprecise stimulation would likely to cause the areas adjacent to the DLPFC to receive the same stimulation. The maintenance of working memory was involved in the network of multiple brain regions, such as the DLPFC and PPC (Curtis, 2006; Ku et al., 2015a; Vogel, McCollough & Machizawa, 2005; Xu & Chun, 2005). Our results seemed to be consistent with the tDCS research on right PPC, in which the authors verified that cathodal tDCS, rather than anodal tDCS, enhanced the precision of working memory for low-baseline performers, for the reason that cathodal tDCS can prevent memory trace from interference (Heinen et al., 2016). Besides, cathodal tDCS can act like a noise filter to enhance cognitive performance in a high-load scene (Weiss & Lavidor, 2012). Therefore, combining the effects of cathodal tDCS on PPC and the results of our study, we suggested that the inhibition control of task-irrelevant information was the main underlying mechanism and tDCS over the right DLPFC could directly regulate the performance in the maintaining process.

However, anodal tDCS over the right DLPFC showed a suppressive rather than facilitative effect on the performance of working memory in our study. Participants tended to show a faster response with a lower hit rate in the difficult condition of the 2-back task. This “trade-off” phenomenon between response time and hit rate might result from the impulsive state of subjects. Subjects showed a tendency to be impulsive not only in the maintaining process but also the whole procedure. We speculated that the speed-accuracy trade-off effects seemed to be induced by the deficit of executive control functions. Due to the sufficient resting time between each block, subjects were able to adjust their strategies to face different upcoming “*n*” conditions. Thereupon, they became more cautious in the difficult condition, which can be manifested by the non-negative effects on the criterion. Nevertheless, anodal tDCS still caused a decreased performance of visuo-spatial working memory. It was speculated that the impulsivity might result from the deficit of working memory, as the result of the hyperpolarization of anodal tDCS over the right DLPFC. Boggio et al. (2010) found that anodal tDCS over the right DLPFC would increase the propensity to risk-taking. Furthermore, previous studies have found that risk-tasking behavior was inversely related to performance of working memory (Romer et al., 2011; Shamosh et al., 2008). A hypothesis has been proposed by Tarter and his colleagues (Tarter et al., 2003), stating that the syndrome of early externalizing behavior as well as poor executive control functions might be the source of early risk-taking. These studies have provided supporting evidences to our hypothesis that the hyperpolarization of anodal tDCS over right DLPFC might cause deficits in executive control functions, which would further lead to the increment of impulsive behavior. Moreover, the disruption of the DLPFC might lead to the decrease of inhibitory control (Romer et al., 2009). Consequently, task-irrelevant response would become difficult to control due to the impulsive interference of working memory performance. Based on the above, it can be summarized that the negative effects of anodal tDCS attributed to the decrease of performance in maintenance.

However, the discriminability index showed no difference in the anodal condition, which indicates that the performance of updating and maintenance was not related to the impulsive behavior. This result could be explained by the findings from Romer et al.’s (2009) study that, although one measure of executive control was highly related to working memory, it cannot contribute to the prediction of overall working memory capacity. Therefore, the study failed to discover the relation between cognitive control (stroop and flanker tasks) and impulsivity. Some other studies also failed to find the convincing relation between risk-taking and executive control in adolescents by employing IGT (Hooper et al., 2004). Thus, the performance of updating and maintenance cannot reflect the deficit of overall executive control functions caused by anodal tDCS, which was tightly associated with impulsivity in our study.

## Conclusion

In sum, the current study attempts to refine the tDCS effects on the sub-processes of working memory. The main findings demonstrate that tDCS can result in different effects at different phases of working memory in the spatial *n*-back task. The tDCS selectively affects the maintenance but not executive manipulation (updating), which suggests that the right DLPFC may not be a special region for updating. To our knowledge, this is the first study that has explicitly investigated how tDCS, respectively, affects the functional sub-processes of working memory. Furthermore, a reversed polarity-specific result was obtained in the present study, showing that cathodal tDCS over the right DLPFC facilitated working memory capacity, whereas anodal tDCS tended to cause an overall impairment of working memory performance. The results suggest that the stimulation of the right DLPFC directly modulates the underlying mechanism by inhibiting task-irrelevant information. Such modulation can influence the performance of working memory, but cannot change the capacity of working memory itself. This study will provide a comprehensive understanding of the tDCS modulation of the sub-processes of working memory and redefine the functional significance of the right DLPFC in working memory and other related therapeutic applications.

## Supplemental Information

10.7717/peerj.4906/supp-1 Supplemental Information 1 Experimental data.

## Additional Information and Declarations

### Data Availability

The following information was supplied regarding data availability: The experimental raw data are provided in a Supplemental File.
